# YUCCA2 (YUC2)-Mediated 3-Indoleacetic Acid (IAA) Biosynthesis Regulates Chloroplast RNA Editing by Relieving the Auxin Response Factor 1 (ARF1)-Dependent Inhibition of Editing Factors in *Arabidopsis thaliana*

**DOI:** 10.3390/ijms242316988

**Published:** 2023-11-30

**Authors:** Zi-Ang Li, Yi Li, Dan Liu, David P. Molloy, Zhou-Fei Luo, Hai-Ou Li, Jing Zhao, Jing Zhou, Yi Su, Ruo-Zhong Wang, Chao Huang, Lang-Tao Xiao

**Affiliations:** 1 Hunan Provincial Key Laboratory of Phytohormones and Growth Development, Hunan Agricultural University, Changsha 410128, China; 602023300026@smail.nju.edu.cn (Z.-A.L.); lixiaoyi@stu.hunau.edu.cn (Y.L.); 2022216045@njau.edu.cn (D.L.); zhoufeiluo@hunau.edu.cn (Z.-F.L.); lihaiou@hunau.edu.cn (H.-O.L.); zhj88717@live.cn (J.Z.); zhoujing@hunau.edu.cn (J.Z.); yisu@hunau.edu.cn (Y.S.); wangruozhong@hunau.edu.cn (R.-Z.W.); 2 Department of Basic Medicine, Chongqing Medical University, Chongqing 400016, China; 190495@cqmu.edu.cn

**Keywords:** YUC2, auxin, chloroplast, RNA editing, ARF1

## Abstract

Although recent research progress on the abundant C-to-U RNA editing events in plant chloroplasts and mitochondria has uncovered many recognition factors and their molecular mechanisms, the intrinsic regulation of RNA editing within plants remains largely unknown. This study aimed to establish a regulatory relationship in *Arabidopsis* between the plant hormone auxin and chloroplast RNA editing. We first analyzed auxin response elements (AuxREs) present within promoters of chloroplast editing factors reported to date. We found that each has more than one AuxRE, suggesting a potential regulatory role of auxin in their expression. Further investigation unveiled that the depletion of auxin synthesis gene *YUC2* reduces the expression of several editing factors. However, in *yuc2* mutants, only the expression of *CRR4*, *DYW1*, *ISE2*, and *ECD1* editing factors and the editing efficiency of their corresponding editing sites, *ndhD*-2 and *rps14*-149, were simultaneously suppressed. In addition, exogenous IAA and the overexpression of *YUC2* enhanced the expression of these editing factors and the editing efficiency at the *ndhD*-2 and *rps14*-149 sites. These results suggested a direct effect of auxin upon the editing of the *ndhD*-2 and *rps14*-149 sites through the modulation of the expression of the editing factors. We further demonstrated that ARF1, a downstream transcription factor in the auxin-signaling pathway, could directly bind to and inactivate the promoters of *CRR4*, *DYW1*, and *ISE2* in a dual-luciferase reporter system, thereby inhibiting their expression. Moreover, the overexpression of *ARF1* in *Arabidopsis* significantly reduced the expression of the three editing factors and the editing efficiency at the *ndhD*-2 and *rps14*-149 sites. These data suggest that YUC2-mediated auxin biosynthesis governs the RNA-editing process through the ARF1-dependent signal transduction pathway.

## 1. Introduction

Chloroplasts serve as solar-powered metabolic factories, playing a crucial role in the biosynthesis and storage of metabolites in higher plants that are essential for sustaining life on Earth [1,2]. Chloroplasts are unique plastids that contain distinct DNA (cpDNA), and although they are semi-autonomous in gene expression [3], they require both nuclear- and plastid-encoded RNA polymerases (NEPs and PEPs, respectively) [4]. PEP predominates in mature chloroplasts and transcribes over 80% of chloroplast genes, while NEP primarily transcribes regulatory genes [5]. After transcription, chloroplast RNAs (cpRNAs) undergo a multitude of post-transcriptional processing events, mainly including RNA processing, editing, and splicing [3,6,7]. In angiosperm plants, C-to-U RNA editing is the most common form, which can produce a new start (ACG-to-AUG) or stop (CAG to UAG, CAA to UAA, or CGA to UGA) and might also impact amino acid coding sequences (CDS) [8,9]. RNA-editing events are considered to be mediated by editosomes of nuclear-encoded editing factors such as pentatricopeptide repeat (PPR) proteins and non-PPR proteins like MORF proteins and ORRM proteins [8]. PPRs are the specificity factors for editing at cytosine sites [10,11,12]. In one example, CRR4, a PPR protein and the first editing factor discovered in plants, regulates the editing of the *ndhD*-2 site by interacting with another PPR protein, DYW1 [12,13]. In addition, several non-PPR proteins, for example, multiple organellar RNA editing factors (MORFs), organelle RNA recognition motif proteins (ORRMs), organelle zinc-finger 1 (OZ1) proteins, protoporphyrinogen oxidase 1 (PPO1), and porphobilinogen deaminase (HEMC), a chloroplast RNA helicase increased size exclusion limit 2 (ISE2), are also required for mediating RNA editing [14,15,16,17,18,19]. These edited genes play important roles in chloroplasts, such as the *ndhD* gene, which encodes a subunit of NADPH dehydrogenase complex on the thylakoid membrane, while *rps14* encodes a subunit of the chloroplast ribosome [13,20].

The primary plant hormone auxin, 3-indoleacetic acid (IAA), plays a crucial role in various plant processes, including growth, development, and the response to environmental stresses [21,22,23,24]. In *Arabidopsis thaliana* (*Arabidopsis*), the IAA biosynthesis pathway involves proteins expressed from the *YUCCA* (*YUC*) gene family [25,26], comprising 11 *YUCs* with distinct expression patterns. *YUC1*, *YUC4*, *YUC10*, and *YUC11* are expressed in embryos [21]; *YUC2* is expressed mainly in young leaves; and *YUC6* is expressed in hypocotyl adventitious root primordials [27,28]. Additionally, expression from *YUC3*, *YUC5*, *YUC7*, *YUC8*, and *YUC9* genes is mainly observed in root tissues, including the proximal meristem and root caps in *Arabidopsis* seedlings [29,30].

The response of plant cells to auxin is primarily mediated through the regulation of gene transcription, which depends on the TIR1/AFB-AUX/IAA-ARF nuclear signaling module [31]. Auxin signaling encompasses both the activation and repression of gene expression through a group of 23 auxin response factor (ARF) genes within the *Arabidopsis* genome [32], of which 22 active ARF proteins interact at auxin response elements (AuxREs) within the promoters of auxin-responsive genes [24,32,33] at canonical 5′-TGTCTC-3′ and 5′-TGTCNN-3′ variable sequences that reflect the differential specificity of regulation between AuxRE isoforms [34,35]. ARF protein activation and the repression of gene transcription relates to a central domain within the transcription factor [32,36]. For example, ARF1 is a repressor of transcription reported to be closely related to YUC2 in regulating the expression of auxin-related genes [37]. Previous studies have uncovered a regulatory relationship between auxin and various processes in chloroplasts, with most of the research focused on the auxin regulation of NEP and PEP polymerases to control chloroplast gene transcription [38,39,40]. However, it remains unclear whether auxin is involved in the post-transcriptional regulatory processes of chloroplast precursor RNA [41,42].

In this study, we investigated the effects of the loss of function of the auxin biosynthesis gene *YUC2* on the expression of chloroplast RNA editing factors and the editing efficiency of chloroplast RNA-editing sites. We found that only the three editing factors CRR4, DYW1, and ISE2, along with their corresponding sites *ndhD*-2 and *rps14*-149, were directly regulated by auxin in early chloroplast development and characterized an ARF gene, *ARF1*. We confirmed that the ARF1-mediated auxin signaling pathway is involved in regulating the expression of *CRR4*, *DYW1*, and *ISE2*, thus affecting the editing efficiency of the *ndhD*-2 and *rps14*-149 sites. These results expand our understanding of the mechanism of auxin in the regulation of chloroplast RNA editing.

## 2. Results

### 2.1. AuxREs in the Promoters of Chloroplast Editing Factors

Auxin-regulatory elements, known as AuxREs, which are necessary for auxin signaling, were found in the promoters of several chloroplast editing proteins, suggesting a possible connection between auxin signaling and chloroplast RNA editing. In order to provide an overview of this possibility, we looked into the AuxREs in the promoters of 42 known *Arabidopsis* chloroplast RNA-editing factors. Sequences located between 1000 and 2000 bp upstream from the ATG start codon for editing factor genes were downloaded from the *Arabidopsis* TAIR10.1 reference genome (https://www.arabidopsis.org/download/index.jsp, accessed on 23 November 2021), and bioinformatics analyses of AuxREs were performed using SnapGene (Ver. 6.0.2). We found that promoters within all genes scrutinized contained two or more core AuxREs, of sequence 5′-TGTC-3′ (Table 1). In addition, more than half of the genes also encompassed variant potential AuxREs of the sequence TGTCTC or TGTCGG within the promoter region (Table 1). The detection of these promoter-region AuxREs in editing factor-encoding genes indicates that auxin may be involved in the regulation of chloroplast RNA-editing factors.

### 2.2. Loss of YUC2 Affects the Expression of Chloroplast Editing Factors

**Figure 1:**
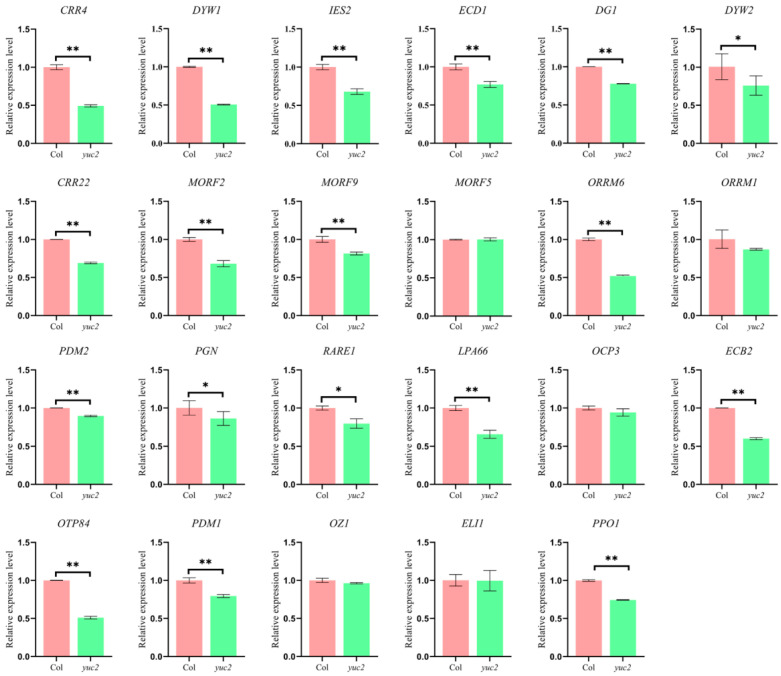
RT-qPCR analysis of the expression levels of chloroplast RNA editing factors in *yuc2*. Values are means ± SD of three replicates; the differences between samples were tested using *t*-test analysis. The two-stage step-up method of Benjamini, Krieger, and Yekutieli was used to correct the *p*-values of the multiple tests performed (desired FDR = 1%) [46], and * above the columns indicates 0.01 < *p* < 0.05, and ** above the columns indicates *p* < 0.01.

*YUC2* encodes a rate-limiting enzyme for auxin synthesis in *Arabidopsis* [43]. To further investigate whether auxin regulates the expression of chloroplast-editing factors, we studied a homozygous T-DNA insertion line of *YUC2* (Figure S1A). Real-time quantitative RT-qPCR data confirmed that *YUC2* gene expression was significantly reduced in the T-DNA insertion mutants (Figure S1B). Considering that auxin plays a role in regulating the early developmental process from the proplastid to the chloroplast, RNA editing can be one of the key processes for normal chloroplast development [44,45]. We analyzed the expression of 23 editing factor genes containing TGTCTC AuxREs in *yuc2*. After 6 h de-etiolation treatment, in comparison to Columbia-0 (Col), RT-qPCR assay results showed that the expression of most editing factors was significantly lower in *yuc2* seedlings than in Col, especially for *CRR4*, *DYW1*, *IES2*, *ECD1*, *DG1*, *CRR22*, *MORF2*, *MORF9*, *ORRM6*, *LPA66*, *ECB2*, *OTP84*, and *PPO1* (Figure 1), suggesting that these genes were transcriptionally regulated by auxin.

### 2.3. RNA-Editing Efficiency of the rps14-149 and ndhD-2 Sites Are Decreased in yuc2

**Figure 2:**
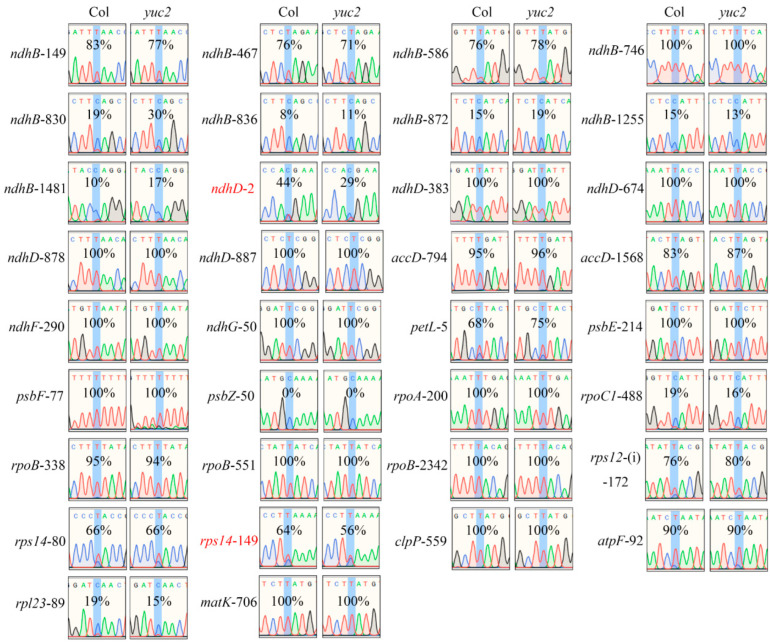
RNA-editing efficiency of the 34 known chloroplast sites in *yuc2* and Col. The editing efficiency of each site was determined by measuring the relative peak height of the nucleotide in the corresponding chromatograms and calculating the percentage of the size of “T” concerning the sum of the height of “T” and “C”.

Considering that the expression of many editing factors was decreased in *yuc2*, we evaluated the editing efficiency of 34 known RNA-editing sites in chloroplast transcripts using Sanger sequencing, which revealed a significant decrease in the editing efficiency of the *ndhD*-2 and *rps14*-149 sites in *yuc2* mutant seedlings compared to Col (Figure 2). However, no noticeable difference in the editing efficiency was observed between *yuc2* mutants and Col at the other 32 sites. Owing to the involvement of the editing factors CRR4 and DYW1 in the editing of *ndhD*-2, as well as the participation of ISE2 and ECD1 in the editing of *rps14*-149, the reduced editing efficiency observed in the *ndhD*-2 and *rps14*-149 sites is consistent with the down-regulation of *CRR4*, *DYW1*, *ISE2*, and *ECD1* expression (Figure 1). Overall, these observations indicate that the editing of *ndhD*-2 and *rps14*-149 is regulated by auxin. We also noted little or no difference in the editing efficiency of the *ndhD*-2 and *rps14*-149 sites in the rosette leaves of adult *yuc2* mutant plants compared to Col (Figure S2). This finding implies that auxin specifically regulates the editing of *ndhD*-2 and *rps14*-149 during early chloroplast development.

### 2.4. Auxin Positively Regulates the Editing of the rps14-149 and ndhD-2 Sites and Their Corresponding Editing Factors

**Figure 3:**
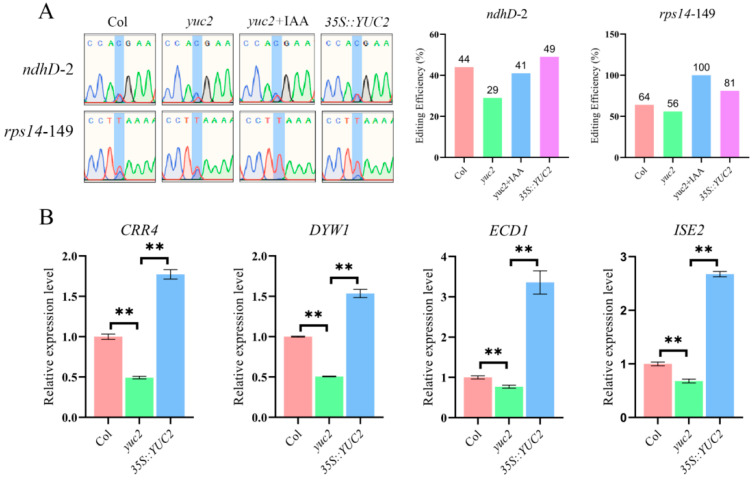
Auxin positively regulates the RNA editing at the *ndhD*-2 and *rps14*-149 sites. (**A**) The application of IAA and the overexpression of *YUC2* led to increased RNA editing efficiency at the *ndhD*-2 and *rps14*-149 sites. (**B**) RT-qPCR detected the expression levels of *CRR4*, *DYW1*, *ISE2*, and *ECD1* in *yuc2* mutants and the YUC2-overexpressing plants. Values are means ± SD of three replicates; the difference between samples was tested using multiple *t*-test analyses. The two-stage step-up method of Benjamini, Krieger, and Yekutieli was used to correct the *p* values of the tests performed (desired FDR = 1%) [46], and ** above the columns indicates *p* < 0.01.

To verify whether the editing efficiency of the *rps14*-149 and *ndhD*-2 sites is directly regulated by auxin, we first evaluated the effects of exogenous auxin treatment on the editing efficiency of these two sites in *yuc2*. The application of IAA significantly increased the editing efficiency of the *rps14*-149 and *ndhD*-2 sites (Figure 3A). Next, we generated *YUC2*-overexpression lines in the *yuc2* mutant background (Figure S1B,C) to validate the connection between editing efficiency and auxin in vivo. The overexpression of *YUC2* led to an increase in editing efficiency at both *rps14*-149 and *ndhD*-2 sites (Figure 3A). We also analyzed the expression of *CRR4*, *DYW1*, *ISE2*, and *ECD1* editing factors in the *YUC2*-overexpressing transgenic plants using RT-qPCR. We found that, in contrast to Col and *yuc2*, the expression levels of the PPR proteins, CRR4 and DYW1, responsible for editing at the *ndhD*-2 site, as well as the ISE2 and ECD1, responsible for editing at the *rps14*-149 site, were increased in *YUC2* overexpressed plants (Figure 3B). These findings suggest that YUC2-mediated auxin synthesis influences the RNA editing at the *ndhD*-2 and *rps14*-149 sites by regulating the expression of *CRR4*, *DYW1*, *ISE2* and *ECD1*.

### 2.5. ARF1 Directly Regulates the Transcription of CRR4, DYW1, and ISE2

**Figure 4:**
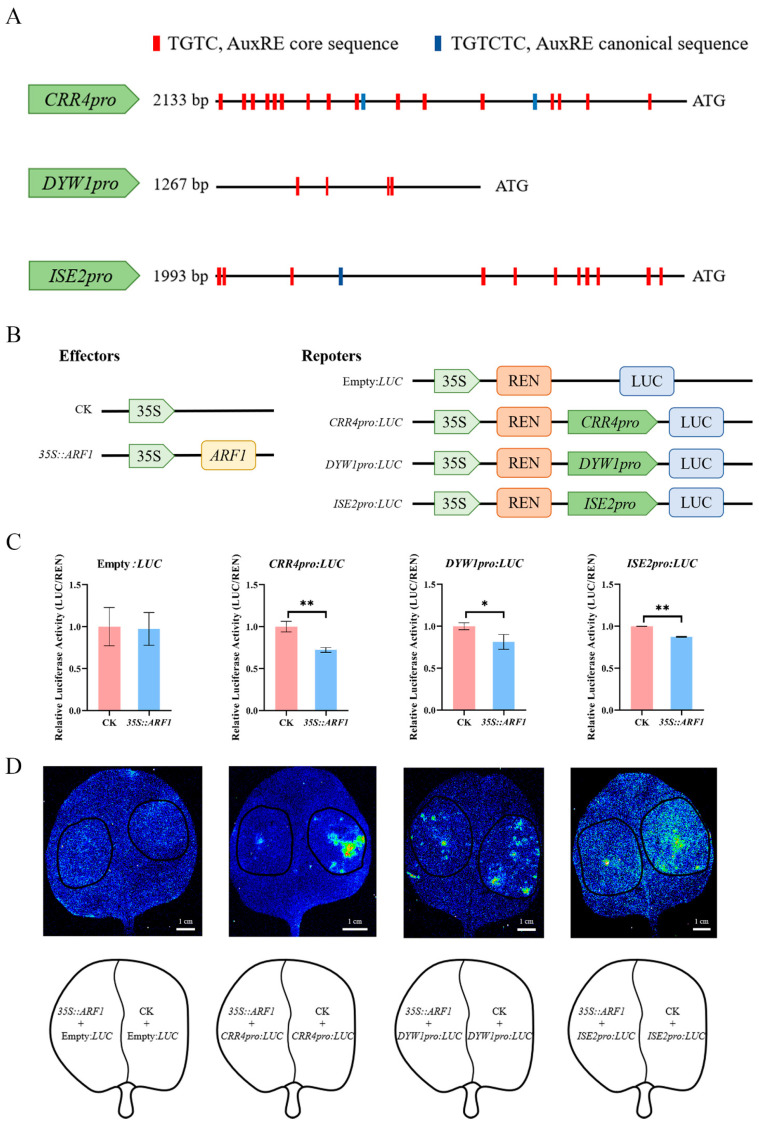
The transcription of *CRR4*, *DYW1*, and *ISE2* is repressed by ARF1. (**A**) Schematic illustrating the positions and quantities of AuxREs within the promoters of *CRR4*, *DYW1*, and *ISE2*. (**B**) Schematic illustrating the construction of effector and reporter vectors. 35S: CaMV 35S promoter. REN: luciferase gene from the anthozoan coelenterate Renilla reniformis. LUC: firefly luciferase gene. (**C**) A transient dual-luciferase assay demonstrated that ARF1 suppressed the transcription of *CRR4*, *DYW1*, and *ISE2* in *Arabidopsis* leaf protoplasts. The reporter genes *CRR4*, *DYW1*, and *ISE2* were co-transformed with effector *35S::ARF1*, respectively. The empty vector pGreenII62SK was used as the negative control. Values are means ± SD of three replicates, the difference between samples was tested by *t*-test analysis. The two-stage step-up method of Benjamini, Krieger, and Yekutieli was used to correct the *p* values of the multiple tests performed (desired FDR = 1%) [46], and * above the columns indicates 0.01 < *p* < 0.05, and ** above the columns indicate *p* < 0.01. (**D**) The luciferase luminescence image of *N. benthamiana* leaves co-infiltrated with the agrobacterial strains containing CRR4pro::LUC, DYW1pro::LUC and ISE2pro::LUC with the 35S::ARF1 effector, respectively. Fluorescence imaging and intensity were conducted using a live-plant-imaging system. The black circles represent injection areas, and the blue color represents the leaves. Scale bar = 1 cm.

Auxin acts on gene expression through its downstream ARF transcription factors that directly bind to the AuxREs elements within the promoter region. Previous research revealed that ARF1 and ARF2 exhibit functional interchangeability and, as principal factors, are involved in the regulation of leaf development via the auxin changes mediated by YUC2 [37,47]. Considering that the promoter regions of the *CRR4*, *DYW1*, and *ISE2* genes contain numerous AuxREs (Figure 4A) demonstrated to be bound by ARF1, we then analyzed the relationship between ARF1 and promoter regions of these genes. A luciferase reporter assay was conducted to investigate the binding of ARF1 to the promoters of *CRR4* (*CRR4pro*), *DYW1* (*DYW1pro*), and *ISE2* (*ISE2pro*). In *Arabidopsis* leaf protoplasts, a transient expression assay revealed that ARF1 effectively repressed the expression of the *CRR4*, *DYW1*, and *ISE2* promoters fused with the *LUC* reporter gene compared to the control. In addition, we co-infiltrated *Nicotiana benthamiana* (*N. benthamiana*) leaves with *Agrobacterium tumefaciens* (*Agrobacterium*) cultures that carry *LUC* reporters and *35S::ARF1* as an effector (Figure 4B). Subsequently, we detected luminescence signals in *N. benthamiana* leaves and observed that the transient expression of ARF1 led to a significant reduction in luminescence signal intensity in leaves expressing CRR4pro::LUC, DYW1pro::LUC, and ISE2pro::LUC (Figure 4C), which is consistent with the results from protoplasts (Figure 4D). These findings indicate that ARF1 functions as a transcriptional repressor by binding to the promoters of *CRR4*, *DYW1*, and *ISE2*, thereby inhibiting gene transcription.

### 2.6. The Deletion of ARF1 Affects the Editing of the ndhD-2 and rps14-149 Sites

**Figure 5:**
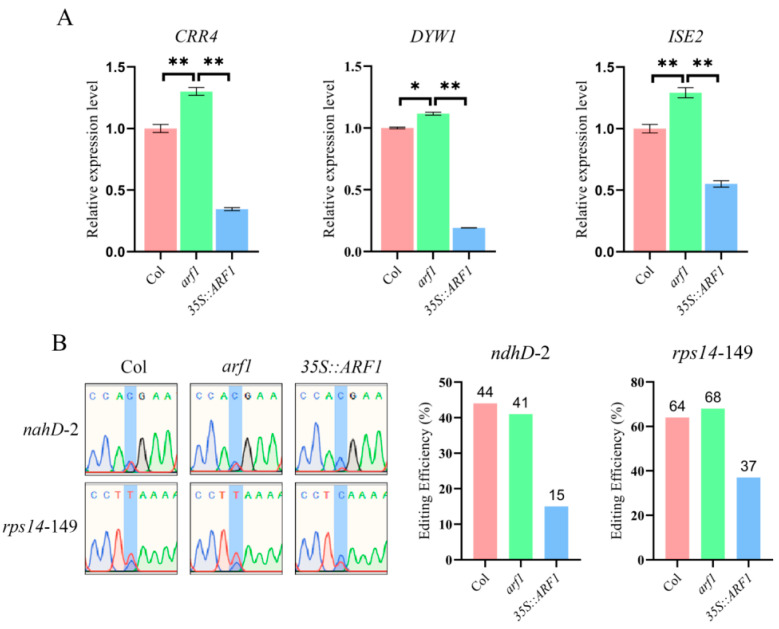
ARF1 regulates the RNA editing at the *ndhD*-2 and *rps14*-149 sites. (**A**) The RNA-editing efficiency of the *ndhD*-2 and *rps14*-149 sites in *arf1* mutants and the ARF1-overexpressing plants. (**B**) RT-qPCR detected the expression levels of *CRR4*, *DYW1*, and *ISE2*. The expression levels of these detected genes were observed to be higher in *arf1* mutants but lower in ARF1-overexpressing plants. Values are means ± SD of three replicates; the difference between samples was tested via multiple *t*-test analyses. The two-stage step-up method of Benjamini, Krieger, and Yekutieli was used to correct the *p*-values of the tests performed (desired FDR = 1%) [46], and * above the columns indicates 0.01 < *p* < 0.05, and ** above the columns indicate *p* < 0.01.

The data above show that ARF1 could directly bind to the promoters of *CRR4*, *DYW1*, and *ISE2* and regulate their expression in *Arabidopsis* protoplasts and tobacco (Figure 4). To illustrate this regulatory relationship further, we analyzed T-DNA line mutant *arf1* and its complemented line using the *ARF1*-overexpression (Figure S3). RT-qPCR results indicated that the accumulation of *CRR4*, *DYW1*, and *ISE2* transcripts was slightly increased in the *arf1* mutants but significantly suppressed in the *ARF1*-overexpressing transgenic plants compared to Col (Figure 5A), confirming that ARF1 negatively regulated these genes. We also tested whether the altered expression of *CRR4*, *DYW1*, and *ISE2* would affect the RNA editing efficiency of the corresponding sites. We observed that the editing of the *rps14*-149 is slightly increased in the absence of *ARF1*. In addition, the editing rate of both the *ndhD*-2 and *rps14*-149 sites was significantly reduced in the ARF1-overexpressing plants, along with the reduced expression of *CRR4*, *DYW1*, and *ISE2* (Figure 5B). Overall, these data suggest that ARF1 can regulate chloroplast RNA editing by suppressing the expression of RNA-editing factors.

## 3. Discussion

**Figure 6:**
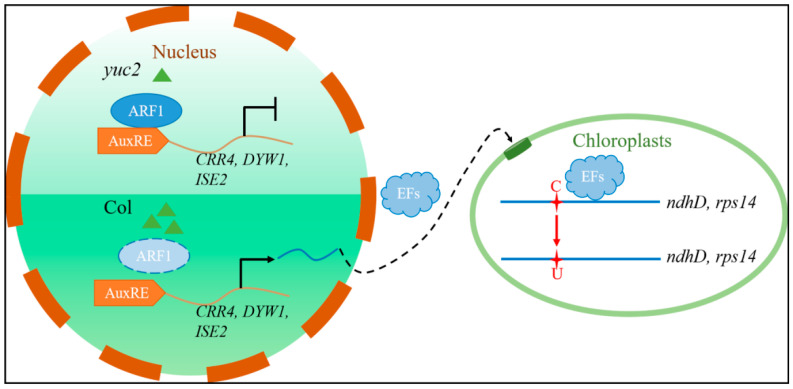
Schematic model of the YUC2-mediated regulation of chloroplast RNA editing through ARF1. Green triangle: IAA molecule; AuxRE: auxin response element; brown curve: genome sequence of editing factor; blue curve: mRNA of editing factors; EFs: editing factor proteins; dark green column: chloroplast protein transport complex; red stars: edited C and T bases; blue lines: edited chloroplast transcripts. In conditions of low auxin concentration (*yuc2*), active ARF1 binds to AuxREs within the promoters of chloroplast RNA editing factors (EFs), such as CRR4, DYW1, and ISE2, resulting in the suppression of these EFs’ expression. Conversely, under circumstances of high auxin levels (Col), the ubiquitination and degradation of Aux/IAA proteins lead to the release of activated ARF1 from the promoters of EFs, thereby promoting the expression of EFs. Following this, the transcribed factors are translated in the cytoplasm and subsequently transported into the chloroplast matrix through transport complexes located on the chloroplast membrane. Within the chloroplast, they carry out RNA editing at the specific *ndhD*-2 and *rps14*-149 sites.

Indole-3-acetic acid is a crucial plant hormone required for growth, development, and chloroplast gene transcription [41,42], although the relationship between auxin and post-transcriptional processes, specifically chloroplast RNA editing, remains elusive. Our present work employed bioinformatics analyses of well-known editing-factor promoters and uncovered an array of AuxREs (Table 1). This intriguing finding suggests that auxin may regulate chloroplast RNA editing. Subsequently, we employed molecular biology, biochemistry, and genetics techniques to study the role of auxin in RNA editing during early chloroplast development in *Arabidopsis*. Significantly, the present work revealed a novel pathway in which *YUC2* regulates chloroplast RNA editing by mediating the expression of editing factors through the downstream ARF1 transcription factor (Figure 6).

In *yuc2* mutant plant de-etiolated seedlings, we noted that significantly reduced editing efficiency at the *ndhD*-2 and *rps14*-149 sites (Figure 2) can be recovered to wild-type levels with the overexpression of *YUC2* and the application of exogenous IAA (Figure 3A). These findings suggest that *YUC2* positively regulates chloroplast RNA editing during early chloroplast development. Intriguingly, no such differences occurred in the editing efficiency at the *ndhD*-2 and *rps14*-149 sites in the mature rosette leaves of *yuc2* (Figure S2). This finding may be attributed to close association of the respective genes with early chloroplast development. The *ndhD* gene encodes for chloroplast NADPH dehydrogenase required for the conversion of protochlorophyllide to chlorophyllide during early chloroplast development. Upon the exposure of etiolated plants to light, NADPH protochlorophyllide oxidoreductase (POR) is photoactivated and catalyzes the conversion of protochlorophyllide to chlorophyllide, which is subsequently esterified into mature chlorophyll [38]. Interestingly, the *ndhD*-2 site is unedited in non-photosynthetic tissues [48], but during photoautotrophism, a process dependent upon elevated levels of chlorophyll synthesis, editing at *ndhD*-2 is completed, reflecting its significance in photosynthetic development. The suppression of *ISE2*, the RNA helicase involved in editing the *rps14*-149 site, also severely disrupts chloroplast development [49]. Furthermore, ECD1 (synonymous with EMB2261) is required to edit the *rps14*-149 site in *Arabidopsis* and is indispensable for early chloroplast development [20]. The silencing of *ECD1* has been linked to *rps14*-149, resulting in the severe impairment of early chloroplast development represented by pronounced albino phenotypes and embryo whitening [20,50]. These studies have demonstrated the critical role of the *ndhD*-2 and *rps14*-149 sites in early chloroplast development. In addition, previous research has shown auxin’s role in regulating plastid-to-chloroplast transition [44]. These data suggest that the specific regulation of editing at these two sites by auxin is feasible during early chloroplast development. 

We also uncovered the regulatory pathway of the YUC2-mediated auxin biosynthesis in chloroplast RNA editing. We demonstrated that the transcriptional repressor ARF1 directly binds to the promoters of *CRR4*, *DYW1*, and *ISE2* to suppress their expression, consequently influencing chloroplast RNA editing (Figure 4C,D and Figure 5B). However, we observed that the editing efficiency at the *ndhD*-2 and *rps14*-149 sites, along with the expression of *CRR4*, *DYW1*, and *ISE2*, showed only minor changes in the *arf1* mutant (Figure 5). In contrast, the significant inhibition of editing efficiency at these two sites and the expression of these three editing factors was observed when *ARF1* was overexpressed. These findings indicate that ARF1 participates in the regulation of *CRR4*, *DYW1*, and *ISE2*, which might be additionally regulated by as-yet-unidentified partner ARFs that imply that a hierarchical complexity exists during the auxin-mediated regulation of RNA editing efficiency, which await future investigations.

## 4. Materials and Methods

### 4.1. Plant Materials and Growth Conditions

The *Arabidopsis* T-DNA insertion lines for *yuc2* (SALK_030199C) and *arf1* (SALK_079046) were obtained from the Arabidopsis Biological Resource Centre. The Col ecotype was used as the wild-type control. T-DNA homozygous plants were selected through PCR genotyping using the primers described in Table S1. Seeds were surface-sterilized with 75% ethanol for 15 min, followed by three rinses with sterilized water. After sterilization, the seeds were planted on 1/2 Murashige and Skoog (MS) medium (Coolaber, Beijing, China) supplemented with 1% (*w*/*v*) sucrose (Coolaber, Beijing, China) and 0.7% agar (Solarbio, Beijing, China), pH 5.8. They were then stratified at 4 °C for 3 days. The seedlings were subsequently cultivated in the dark at 22 °C for 7 days. The seedlings were exposed to experimental light conditions at 22 °C for 6 h for the de-etiolation treatment.

### 4.2. Constructs and Plant Transformation

To generate the constructs of *35S::YUC2-GFP* and *35S::ARF1-MYC*, the full-length CDS of *YUC2* (1248 bp) and *ARF1* (1998 bp) were amplified using TransStart^®^ FastPfu DNA Polymerase (Transgen, Beijing, China) following the manufacturer’s protocol. The amplification primers are *YUC2* (*Kpn* I)-F and *YUC2* (*Sal* I)-R, as well as *ARF1* (*BamH* I)-F and *ARF1* (*EcoR* I)-R, and the primers sequences are listed in Table S1. The resulting fragments were recovered by *Kpn I/Sal I* and *EcoR I/BamH I* restriction digestion, respectively, and then subcloned into the binary vector pCAMBIA1300 through T4 ligase (Takara, Shiga, Japan), which contains the CaMV 35S promoter. The connected product was transformed into chemically competent *Escherichia coli* DH5α (Weidi, Shanghai, China) using a heat shock method. The transformed cells were plated onto LB agar medium containing kanamycin (50 μg/mL, Yuanye, Shanghai, China) resistance and incubated for approximately 16 h. Positive colonies grown on the plates were subjected to PCR identification and subsequently sent to Qingke company for sequencing to confirm that the vector did not contain any mutations. Subsequently, the constructs were introduced into chemically competent *Agrobacterium* strain GV3101 using a heat shock method and transformed into a mutant background through *Agrobacterium*-mediated floral dipping.

### 4.3. RNA Extraction and RT-qPCR

Total RNA was extracted using the HiPure Plant RNA Mini Kit (Magen, Guangzhou, China) following the manufacturer’s protocol. Following the manufacturer’s protocol, the first-strand cDNA was synthesized using HiScript^®^ III 1st Strand cDNA Synthesis Kit (Vazyme, Nanjing, China). RT-qPCR analyses were performed on a CFX96 Touch Real-Time PCR Detection System (Bio-rad, Hercules, CA, USA) using ChamQ Universal SYBR qPCR Master Mix (Vazyme, Nanjing, China) according to the manufacturer’s instructions. *ACTIN2* (AT3G18780) was used as a reference gene to calculate relative expression values using the 2^−ΔΔCt^ method [51]. The values of each sample are the means ± SD of three replicates. Statistical differences between the samples were tested using mut-test analysis. The two-stage step-up method of Benjamini, Krieger, and Yekutieli was used to correct the *p*-values of the tests performed (desired FDR = 1%). The specific primers for the genes are listed in Table S1.

### 4.4. RNA Editing Efficiency Analysis

First-strand cDNA was synthesized for the analysis of RNA-editing efficiency using HiScript III 1st Strand cDNA Synthesis Kit (Vazyme, Nanjing, China). The PCR products of the corresponding chloroplast and mitochondrial genes were amplified using 2 × Taq Master Mix (Novoprotein, Suzhou, China) with specific primers listed in Table S1. Then, they were subjected to a company (Tsingke, Beijing, China) for Sanger sequencing. The editing efficiency of each site was determined by measuring the relative peak height of the nucleotide in the corresponding chromatograms and calculating the percentage of the size of “T” concerning the sum of the height of “T” and “C” [52].

### 4.5. Luciferase Reporter Assay

According to a previously described protocol, *Arabidopsis* protoplasts were isolated from three-week-old WT leaves [53]. The coding sequence of *ARF1* (1998 bp) was integrated into the pGreenII62SK vector, which was employed as effectors, while the *CRR4pro* (2133 bp), *DYW1pro* (1267 bp), and *ISE2pro* (1993 bp) were inserted into the pGreenII0800-LUC vector for use as reporters. The primers used for luciferase reporter assay are listed in Table S1. The pGreenII62SK-*ARF1* effector plasmid was transferred into *Arabidopsis* protoplast cells with each of the reporter plasmids (*CRR4pro::LUC*, *DYW1pro::LUC*, and *ISE2pro::LUC*). The empty vector pGreenII62SK was used as the negative control. The dual-luciferase assay kit (Promega, Madison, WI, USA) allows for the quantification of both firefly and renilla luciferase activity. The fluorescence signals were detected with a Spark multi-mode plate reader (Tecan, Männedorf, Switzerland).

### 4.6. Transient Expression in N. benthamiana

The reporter and effector vectors constructed above were transformed into GV3101 (with pSoup) (WeiDi, Shanghai, China), respectively, and then co-transferred into the leaves of 1-month-old *N. benthamiana* for transient expression. Briefly, the transformed Agrobacteria were inoculated in LB medium with suitable antibiotics and cultured for over 12 h. Afterwards, we resuspended the *Agrobacterium* of each reporter and effector with the MES buffer and adjusted the OD600 (with 150 mM acetosyringone) to 0.5. The suspensions were then mixed separately to achieve a final OD600 of 1.0 and left at room temperature for 1 h before being injected into tobacco leaves. The infected tobacco plants were cultured for 24 h in darkness and subsequently transferred to a light incubator (25 °C, 16 h light; 8 h dark). After 48 h, the tobacco leaves were photographed using an in vivo real-time molecular marker imager for plants (Lumazone PyLoN1300B, Teledyne Princeton Instruments, Trenton, NJ, USA).

## 5. Conclusions

In this paper, we present, for the first time, the regulatory role of the plant hormone auxin in chloroplast RNA editing and elucidate its precise molecular regulatory mechanism. This narrative highlights the intriguing connection between auxin and chloroplast RNA editing, addressing the fundamental theoretical question of how plants internally regulate chloroplast RNA editing. Furthermore, it expands our understanding of chloroplast RNA editing.

## Figures and Tables

**Table 1:** The number of AuxREs in the promoters of chloroplast RNA-editing factors in *Arabidopsis*.

Gene	Promoter Length	TGTCNN	TGTCTC	TGTCGG
*ATPF editing factor 1* (*AEF1*)	2031	9	0	0
*chloroplast biogenesis 19* (*CLB19*)	822	5	0	0
*chloroplast RNA-binding protein 31a* (*CP31A*)	2096	7	2	0
*chloroplast RNA-binding protein 31b* (*CP31B*)	1935	11	0	0
*chloroplast RNA editing factor 3* (*CREF3*)	1849	18	0	1
*chloroplast RNA editing factor 7* (*CREF7*)	1678	19	1	0
*chlororespiratory reduction 2* (*CRR2*)	1851	11	0	0
*chlororespiratory reduction 2* (*CRR4*)	2143	18	2	0
*chlororespiratory reduction 21* (*CRR21*)	1567	17	1	1
*chlororespiratory reduction 22* (*CRR22*)	1817	19	2	0
*chlororespiratory reduction 28* (*CRR28*)	2216	8	0	0
*delayed greening 1* (*DG1*)	1550	16	1	0
*defectively organized tributaries 4* (*DOT4*)	1149	8	1	1
*dyw domain protein 1* (*DYW1*)	1267	4	0	0
*dyw domain protein 2* (*DYW2*)	2106	16	2	0
*Arabidopsis early chloroplast biogenesis 2* (*ECB2*)	1610	9	2	0
*early chloroplast development 1* (*ECD1*)	2000	10	1	1
*ectopic lignification 1* (*ELI1*)	2162	18	3	0
*hydroxymethylbilane synthase* (*HEMC*)	655	4	0	0
*increased size exclusion limit 2* (*ISE2*)	1993	12	0	0
*low PSII accumulation 66* (*LPA66*)	1291	6	1	0
*mitochondrial editing factor 37* (*MEF37*)	1873	11	1	0
*multiple organellar rna editing factor 2* (*MORF2*)	2073	13	2	0
*multiple organellar rna editing factor 5* (*MORF5*)	1976	6	1	0
*multiple organellar rna editing factor 8* (*MORF8*)	2109	18	2	0
*multiple organellar rna editing factor 9* (*MORF9*)	1872	15	0	0
*NUWA* (named after the well-known goddess in ancient Chinese mythology)	1914	6	0	0
*overexpressor of cationic peroxidase 3* (*OCP3*)	878	3	1	0
*organelle rna recognition motif protein 1* (*ORRM1*)	1938	5	0	0
*organelle rna recognition motif protein 6* (*ORMM6*)	1114	8	2	1
*organelle transcript processing 80* (*OTP80*)	1873	9	0	0
*organelle transcript processing 81* (*OTP81*)	1983	10	0	1
*organelle transcript processing 82* (*OTP82*)	1801	2	0	0
*organelle transcript processing 84* (*OTP84*)	1597	9	1	0
*organelle transcript processing 85* (*OTP85*)	1967	11	2	0
*organelle transcript processing 86* (*OTP86*)	913	5	1	0
*organelle transcript processing 90* (*OTP90*)	2028	12	1	0
*organelle zinc finger 1* (*OZ1*)	2129	10	2	0
*pentratricopeptide repeat protein pigment-defective mutant 1* (*PDM1*)	1797	21	6	2
*pentratricopeptide repeat protein pigment-defective mutant 2* (*PDM2*)	2028	16	1	1
*pentatricopeptide repeat protein for germination on NaCl* (*PGN*)	2202	15	1	1
*protoporphyrinogen ix oxidase 1* (*PPO1*)	2045	19	1	0
*required for accD RNA editing 1* (*RARE1*)	2188	12	1	0
*tRNA arginine adenosine deaminase* (*TADA*)	2075	10	0	0
*white to green 1* (*WTG1*)	1623	11	0	1
*yellow seedling1* (*YS1*)	1266	7	0	0

## Data Availability

Data are contained within the article and Supplementary Materials.

## Supplementary Materials

The following supporting information can be downloaded at https://www.mdpi.com/article/10.3390/ijms242316988/s1.
