# Supplementary material for: YUCCA2 (YUC2)-Mediated 3-Indoleacetic Acid (IAA) Biosynthesis Regulates Chloroplast RNA Editing by Relieving the Auxin Response Factor 1 (ARF1)-Dependent Inhibition of Editing Factors in Arabidopsis thaliana
Source: Int J Mol Sci. 2023 Nov 30;24(23):16988. doi: 10.3390/ijms242316988 (PMC10706925; doi:10.3390/ijms242316988)
Supplement: Supplementary file 1 [file ijms-24-16988-s001.zip › ijms-2684307-supplementary.pdf]

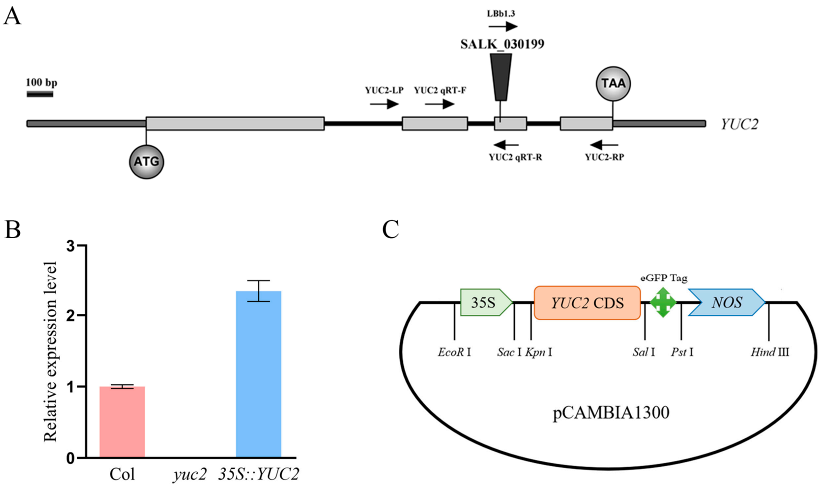

Figure S1. (A) The positions of the T-DNA insertions in the *YUC2* are shown. The colours from light to dark represents the CDS, UTR and intron, respectively. The arrows represent the primers used in researches. (B) The qPCR analysis of the expression of *YUC2* in Col, *yuc2* and over-expressed *YU*C2. (C) Schematic illustration of the *YUC2*-fusion construct. Expression of the YUC2 code sequence fused with eGFP-tag is driven by the CaMV 35S promoter. Transcription is terminated by using the nopaline synthase terminator and poly(A) signal (NOS).

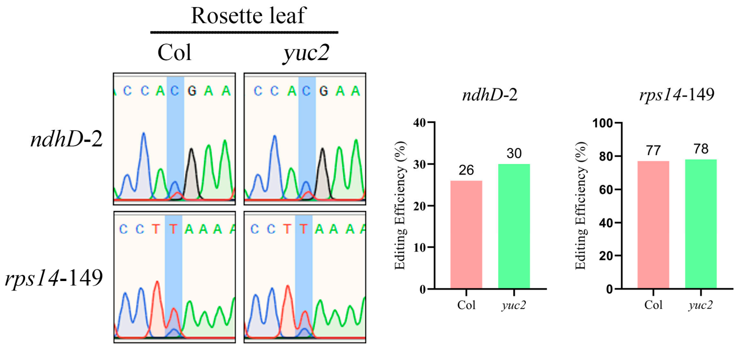

Figure S2. The editing efficiency of the *ndhD*-2 and *rps14*-149 loci in *yuc2* and Col lotus leaves. Seedlings grown in long day environment (16h light and 8h dark) at 22℃ for 4 weeks.

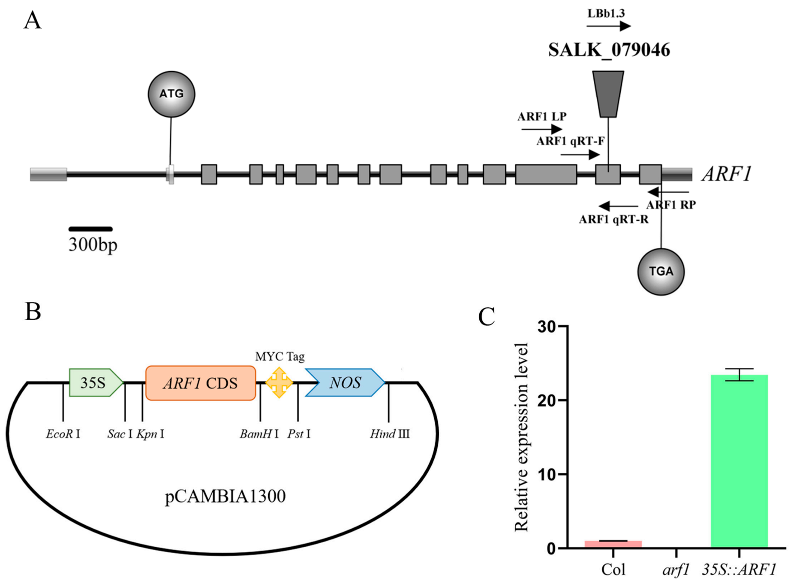

Figure S3. (A) The positions of the T-DNA insertions in the *ARF1* are shown. The colours from light to dark represents the CDS, UTR and intron, respectively. The arrows represent the primers used in researches. (B) Schematic illustration of the *ARF1*-fusion construct. Expression of the *ARF1* code sequence fused with 4×MYC-tag is driven by the CaMV 35S promoter. Transcription is terminated by using the nopaline synthase terminator and poly(A) signal (NOS). (C) The qPCR analysis of the expression of *ARF1* in Col, *yuc2* and over-expressed *YU*C2.

Table S1. List of primers used in this work

Primer name	Used for	Primer sequence (5'-3')
YUC2-LP	Genotyping	TTGTAGGATGTGGAAACTCCG
YUC2-RP	Genotyping	ATAACCCAATCCAAACTTGCC
ARF1-LP	Genotyping	CAGACACTCCTTCCTCAGTGC
ARF1-RP	Genotyping	GCCCTCTGTATTCCCATTTTC
LBb1.3	Genotyping	ATTTTGCCGATTTCGGAAC
YUC2 (Kpn I)-F	Construction	CCGGGTACCATGGAGTTTGTTACAGAAACG
YUC2 (Sal I)-R	Construction	ACGCGTCGACTTAACAATGTTGAGGACGAG
ARF1 (BamH I)-F	Construction	CGCGGATCCATGGCAGCTTCCAATCATTC
ARF1 (EroR I)-R	Construction	CCGGAATTCTCATCTTGATCCCGCCATAG
CRR4pro (Kpn I)-R	Construction	CGGGGTACCCTCTTTGTCGCTCTTACAct
CRR4pro (Xho I)-F	Construction	CCGCTCGAGcagttattacgttcttctgttgg
DYW1pro (Kpn I)-F	Construction	CGGGGTACCCCAAAGCTCCATCATCACCA
DYW1pro (Xho I)-R	Construction	CCGCTCGAGccctttgtggtcaatccaag
ISE2pro (Kpn I)-F	Construction	CGGGGTACCGTGGACATTGTGGACAACA
ISE2pro (Xho I)-R	Construction	CCGCTCGAGgttttcagggcatttcaaga
CRR4 qRT-F	qPCR	TGCCGAGAAGAGATGTAGTTAC
CRR4 qRT-R	qPCR	GCATTTGGTCAAACAGAGTCTT
DG1 qRT-F	qPCR	AGGCAGAAACCGACTAAACTAA

DG1 qRT-R	qPCR	CAAGCGTTAAGAATAGCGTTGT
DYW1 qRT-F	qPCR	AGGTTAGAGGAGATAAGCCAGA
DYW1 qRT-R	qPCR	CGTTCACTATGATGCATCAAGG
ISE2 qRT-F	qPCR	GATGCAGCTGTACAGTATGTTG
ISE2 qRT-R	qPCR	CACTGCATCTGGATAAAGAACG
ECD1 qRT-F	qPCR	CAGAAATAAAACGCTGCGGATA
ECD1 qRT-R	qPCR	CAATTTTCTCGCTGTGCTGATA
DYW2 qRT-F	qPCR	GTCGCTTGAGCATTCTAAGAAG
DYW2 qRT-R	qPCR	TTCCGTTATCACTATACGCACA
MORF2 qRT-F	qPCR	GGTTCTTCTCAATTCGATGTGG
MORF2 qRT-R	qPCR	GAGGCCGATCACTAAAGTTAGA
MORF9 qRT-R	qPCR	GCGAAGAAGAACATGTATGCAT
MORF9 qRT-F	qPCR	GCGAAGAAGAACATGTATGCAT
MORF5 qRT F	qPCR	TCTTATATCCTCTCCCGTCGAT
MORF5 qRT R	qPCR	ATCAACCAATGCTCATAATCGC
ORRM6 qRT-F	qPCR	ATCTAGCTGTCTCTCTACGGAT
ORRM6 qRT-R	qPCR	TCCATCACCATGTTCATGTGTA
ORRM1 qRT-F	qPCR	GTCGAGATTCTTGCGAAAGTAC
ORRM1 qRT-R	qPCR	CTACTCCAGGTAAACTAGCGAG
CRR22 qRT-F	qPCR	AATTACACATCATGCGAAGTGG
CRR22 qRT-R	qPCR	CATAATAGCACCACATTTCGCA
PGN qRT-F	qPCR	TACTCTAATCACCGGGTTTACG
PGN qRT-R	qPCR	CGTTTAATCACAGACGCATGAA
RARE1 qRT-F	qPCR	GAGTTCCGGTTATAAAGAACGC
RARE1 qRT-R	qPCR	GGTAAGAATACGAGCTAACGGA
LPA66 qRT-F	qPCR	AAGATGATTCACACCCAAAAGC
LPA66 qRT-R	qPCR	CTTATCAACCCAAACGCTGTAG
OCP3 qRT-F	qPCR	TCAGGATTATCCGTCAACAGAG
OCP3 qRT-R	qPCR	CACGAGCAAAAACAAGTACAGA
ECB2 qRT-F	qPCR	TCTTCTGCTCAAAGTCCTCATT
ECB2 qRT-R	qPCR	CACAAACACATCTTCGTCTACC
OTP84 qRT-F	qPCR	TTATCTCTCAATCACGCTCTCC
OTP84 qRT-R	qPCR	CAAAGCAGGGAACGCATAATTA
PDM1 qRT-F	qPCR	AGATGTTTTTGTGGGAACTTCG
PDM1 qRT-R	qPCR	CACACGCTCTAAGAATGCTTAC
PDM2 qRT-F	qPCR	AGAACGAGATGCCGTATTGTAT
PDM2 qRT-R	qPCR	CATAAGTGGTTTCAGAGTCCCT
ELI1 qRT-F	qPCR	CACTTGTTTCCAAAGCCCTAAA
ELI1 qRT-R	qPCR	CTTGTATTATTTCCAGGCCACG
PPO1 qRT-F	qPCR	TCTGTCCAAGTCTGAAGGTG
PPO1 qRT-R	qPCR	TGTAAGCGTACCGTGACATG

OZ1 qRT-F	qPCR	GTTTGCCAAGAAACCTAAGGAG
OZ1 qRT-R	qPCR	AGTCTGAAACATTGTGCAGTTC
YUC2 qRT-F	qPCR	CAAGATCAAATGCGGAAAGACT
YUC2 qRT-R	qPCR	CCGAATAATGCATTACCCGTTT
ARF1 qRT-F	qPCR	AAGTAGACAGATACGTAGCTGC
ARF1 qRT-R	qPCR	CTTCATCATCGGTGTAAACGAC
ACTIN2 qRT-F	qPCR	CTCCTTTGTTGCTGTTGACTAC
ACTIN2 qRT-R	qPCR	GCACAATGTTACCGTACAGATC
accD-F1	Editing efficiency	TAGTGAAAGCGGAAAGATTC
accD-R1	Editing efficiency	AATACCGTTTAGTTGACCTG
accD-F2	Editing efficiency	GCATTTGCGGGTAAAAGAGTAA
accD-R2	Editing efficiency	GAATCtgatctaacaacaggga
atpF-F	Editing efficiency	CTATGGTTCTCAAATATGATTT
atpF-R	Editing efficiency	CTAGCTTAATTCTAATAAGTCTCA
clpP-F	Editing efficiency	TTTCGGATTGGCTGTCTTG
clpP-R	Editing efficiency	TATGAGGCACAAACGGGAG
matK-F	Editing efficiency	TTCCATAGAATACAATTCGCTC
matK-R	Editing efficiency	CAATTCATTCAATATTTCCCTT
ndhB-F1	Editing efficiency	GCGGAACCGATCTACTAATTC
ndhB-R1	Editing efficiency	GATCTTATACATACGCGAGG
ndhB-F2	Editing efficiency	AACTACCCGTAATAACAAAC
ndhB-R2	Editing efficiency	GGATCAACTAAGCCCTCT
ndhD-F1	Editing efficiency	GTCCAAAGAACGCGTACTCaa
ndhD-R1	Editing efficiency	CCATCTATTCCCATTCTCCAG
ndhD-R2	Editing efficiency	CCACTTTAGCGGCTTTTCCA
ndhD-F2	Editing efficiency	AACCAACCCATAGGCTCCCA
ndhD-F3	Editing efficiency	TGGGAGCCTATGGGTTGGTT
ndhD-R3	Editing efficiency	GCAATGCAAGGGAAGCCATC
ndhF-F	Editing efficiency	AGCATTCGCTGCAATAGGTCGT
ndhF-R	Editing efficiency	ATCATCCCTTTCATTCCACTTC
ndhG-F:	Editing efficiency	tcacagcaaatatgattaaaac
ndhG-R	Editing efficiency	gagaaaacagcctaatactaat
petL-F	Editing efficiency	GAGATAGTGCAGATGCCAAAGA
petL-R	Editing efficiency	GGAAAGCAAGAAGGAGGTTAGT
psbE-F	Editing efficiency	ACTTTGTTCGTTCGGATTT
psbE-R	Editing efficiency	CTGGGTCATTCATAGCATT
psbF-F	Editing efficiency	ACAGGAGAACGTTCTTTTGC
psbF-R	Editing efficiency	ATATAATCCATCCGAATGGG
psbZ-F	Editing efficiency	GAACTAAGTCATCTCGAATC
psbZ-R	Editing efficiency	TTCAAGTTCCATAAGTTCGAC
rpl23-F	Editing efficiency	CTGGCATTACGACCTTTAC

rpl23-R	Editing efficiency	CTATTGGAATTGGCTCTGT
rpoA-F	Editing efficiency	ACAGCGGGCGGTAAAATAATGT
rpoA-R	Editing efficiency	aaggctaaaagggtttcaagag
rpoB-F1	Editing efficiency	TGCGTGAGGGTACTCCTAATGG
rpoB-R1	Editing efficiency	CGAAAGAATCCTCCTATGCTCC
rpoB-F2	Editing efficiency	GACCAATCCTTCCTAATTCACA
rpoB-R2	Editing efficiency	AAGAACGAGATGCTGTCTATGA
rpoC1-F	Editing efficiency	TTCCCCGTAGGCCCTTCTTCTC
rpoC1-R	Editing efficiency	GAGAGATAGTTGGAGAGGTGAC
rps12-F	Editing efficiency	CATTTACCCTGTTAGTCCG
rps12-R	Editing efficiency	AATACAAGACAGCCAATCC
rps14-F	Editing efficiency	AAACATGCCTGAACCATTTCCC
rps14-R	Editing efficiency	TCCGATTTGTATCATTATCATTG
